# Surgical management of chest injuries in patients with multiple and/or severe trauma– a systematic review and clinical practice guideline update

**DOI:** 10.1007/s00068-024-02556-1

**Published:** 2024-06-18

**Authors:** C. Schreyer, S. Schulz-Drost, A. Markewitz, J. Breuing, B. Prediger, L. Becker, C. Spering, J. Neudecker, B. Thiel, D. Bieler

**Affiliations:** 1 Department of General, Visceral and Thoracic Surgery, German Armed Forces Central Hospital, Koblenz, Germany; 2 Department of Trauma Surgery, Schwerin Helios Hospital, Schwerin and Department of Trauma and Orthopaedic Surgery, Schwerin, Germany; 3 Friedrich-Alexander University Erlangen-Nürnberg (FAU), University Hospital Erlangen, Erlangen, Germany; 4 German Society for Thoracic and Cardiovascular Surgery, Berlin, Germany; 5 Institute for Research in Operative Medicine (IFOM), Witten/Herdecke University, Cologne, Germany; 6 Department of Trauma Surgery, Hand and Reconstructive Surgery, Essen University Hospital, Essen, Germany; 7 Department of Trauma Surgery, Orthopaedics, and Plastic Surgery, Göttingen University Medical Centre, Göttingen, Germany; 8 Department of Surgery, Berlin Charité Hospital, Campus Charité Mitte and Campus Virchow, Berlin, Germany; 9 Department of Thoracic Surgery, Klinikum Westfalen Knappschaft, Lünen, Germany; 10 Department for Trauma Surgery and Orthopaedics, Reconstructive Surgery, Hand Surgery, Burn Medicine, German Armed Forces Central Hospital, Koblenz, Germany; 11 Department for Orthopaedics and Trauma Surgery, Medical Faculty and University Hospital, Heinrich Heine University, Duesseldorf, Germany

**Keywords:** Surgical management, Chest, VATS in thoracic trauma, Lung injury, Tracheobronchial injury, Thoracic aortic rupture, Polytrauma guideline

## Abstract

**Purpose:**

Our aim was to update evidence-based and consensus-based recommendations for the surgical and interventional management of blunt or penetrating injuries to the chest in patients with multiple and/or severe injuries on the basis of current evidence. This guideline topic is part of the 2022 update of the German Guideline on the Treatment of Patients with Multiple and/or Severe Injuries.

**Methods:**

MEDLINE and Embase were systematically searched to May and June 2021 respectively for the update and new questions. Further literature reports were obtained from clinical experts. Randomised controlled trials, prospective cohort studies, cross-sectional studies and comparative registry studies were included if they compared interventions for the surgical management of injuries to the chest in patients with multiple and/or severe injuries. We considered patient-relevant clinical outcomes such as mortality, length of stay, and diagnostic test accuracy. Risk of bias was assessed using NICE 2012 checklists. The evidence was synthesised narratively, and expert consensus was used to develop recommendations and determine their strength.

**Results:**

One study was identified. This study compared wedge resection, lobectomy and pneumonectomy in the management of patients with severe chest trauma that required some form of lung resection. Based on the updated evidence and expert consensus, one recommendation was modified and two additional good practice points were developed. All achieved strong consensus. The recommendation on the amount of blood loss that is used as an indication for surgical intervention in patients with chest injuries was modified to reflect new findings in trauma care and patient stabilisation. The new good clinical practice points (GPPs) on the use of video-assisted thoracoscopic surgery (VATS) in patients with initial circulatory stability are also in line with current practice in patient care.

**Conclusion:**

As has been shown in recent decades, the treatment of chest trauma has become less and less invasive for the patient as diagnostic and technical possibilities have expanded. Examples include interventional stenting of aortic injuries, video-assisted thoracoscopy and parenchyma-sparing treatment of lung injuries. These less invasive treatment concepts reduce morbidity and mortality in the primary surgical phase following a chest trauma.

## Introduction

In Germany, almost half of all polytrauma patients (46.1%) [1] present with severe thoracic trauma. A relevant thoracic trauma can immediately or at a later stage lead to a serious life-threatening condition in the presence of airway injuries (A problem) or to respiratory (B problem) and haemodynamic (C problem) compromise. In German-speaking countries, emergency thoracotomy (within one hour of arrival at the hospital) is necessary in only 0.9% of patients with an Injury Severity Score (ISS) ≥ 9 [2]. Due to improved stabilization measures, interventional and minimally invasive procedures that are gentle on the patient are increasingly being used in the treatment of polytrauma in patients with stable or stabilised vital signs, even when they present with thoracic injuries. The objective of this systematic review is to assess the evidence for current therapeutic and surgical procedures in the initial surgical management of thoracic trauma in the hospital setting.

## Methods

This guideline topic is part of the 2022 update of the German Guideline on the Treatment of Patients with Multiple and/or Severe Injuries [3]. The guideline update is reported according to the RIGHT tool [4], the systematic review part according to the Preferred Reporting Items for Systematic Reviews and Meta-Analyses (PRISMA) 2020 reporting guideline [5]. The development and updating of recommendations followed the standard methodology set out in the guideline development handbook issued by the German Association of the Scientific Medical Societies (AWMF) [6]. All methods were defined a priori, following the methods report of the previous guideline version from July 2016 [7] with minor modifications, as detailed below. The Introduction and Discussion sections of this publication are summaries of the original guideline text [3].

### PICO questions and eligibility criteria

Population, intervention, comparison, and outcome (PICO) questions were retained from the previous guideline version. In addition, the participating professional societies involved in guideline development were asked to submit new PICO questions. The overarching PICO question for this topic area was:

*In adult patients (≥ 14 years) with known or suspected polytrauma and/or severe injuries, does a specific surgical approach to the management of chest injuries improve patient relevant outcomes compared to any other intervention?*

The full set of predefined PICO questions is listed in Table S1 (Online Resource 1). The study selection criteria in the PICO format are shown in Table 1.

**Table 1:** Predefined selection criteria

Population:	Adult patients (≥ 14 years) with polytrauma and/or severe injuries^a^
**Intervention** **/comparison**:	surgical management of injuries to the chest
**Outcomes**:	any patient-relevant outcome such as mortality or length of stay, diagnostic test accuracy
**Study type**:	• comparative, prospective studies (randomised controlled trials, cohort studies) • comparative registry^b^ data (incl. case-control studies) • cross-sectional studies (only diagnostic studies) • systematic reviews based on the above primary study types
**Language**:	English or German
**Other inclusion criteria**:	• full text of study published and accessible • study matches predefined PICO question
**Exclusion criteria**:	• multiple publications of the same study without additional information

^a^ Defined by an Injury Severity Score (ISS) > 15, Glasgow Coma Scale (GCS) < 9, or comparable values on other scales, or, in the prehospital setting, clinical suspicion of polytrauma/severe injury with a need for life-saving interventions

^b^ Using the Agency for Healthcare Research and Quality (AHRQ) definition of registries [8]

### Literature search

An information specialist systematically searched for literature in MEDLINE (Ovid) and Embase (Elsevier). The search strategy described in the 2011 Guideline was used with modifications. It contained index (MeSH/Emtree) and free text terms for the population and intervention. All searches were conducted in May and June 2021 for the update and new questions. The start date for update searches was 8 June 2014. No start date was used in the searches for new PICO questions. Table S2 (Online Resource 1) provides details for all searches. Searches were conducted for both prehospital and inhospital care. Clinical experts were asked to submit additional relevant references.

### Study selection

Study selection was performed independently by two reviewers in a two-step process using the predefined eligibility criteria: (1) title/abstract screening of all references retrieved from database searches using Rayyan software [9] and (2) full-text screening of all articles deemed potentially relevant by at least one reviewer at the title/abstract level in Endnote (Endnote, Version: 20 [Software], Clarivate, Boston, Massachusetts, USA. https://endnote.com/). Disagreements were resolved through consensus or by consulting a third reviewer. The reasons for full-text exclusion were recorded (Table S3, Online Resource 1).

### Assessment of risk of bias and level of evidence

Two reviewers sequentially assessed the risk of bias of included studies at study level using the relevant checklists from the NICE guidelines manual 2012 [10] and assigned each study an initial level of evidence (LoE) using the Oxford Centre for Evidence-based Medicine Levels of Evidence (2009) [11]. Any disagreements were resolved through consensus or by consulting a third reviewer.

### Data extraction and data items

Data were extracted into a standardised data table by one reviewer and checked by another. A predefined data set was collected for each study, consisting of study characteristics (study type, aims, setting), patient selection criteria and baseline characteristics (age, gender, injury scores, other relevant variables), intervention and control group treatments (including important co-interventions, index and reference tests for diagnostic studies), patient flow (number of patients included and analysed), matching/adjusting variables, and data on outcomes for any time point reported.

### Outcome measures

Outcomes were extracted as reported in the study publications. For prospective cohort studies and registry data, preference was given to data obtained after propensity-score matching or statistical adjustment for risk-modulating variables over unadjusted data.

### Synthesis of studies

Studies were grouped by interventions. An interdisciplinary expert group used their clinical experience to synthesise studies narratively by balancing beneficial and adverse effects extracted from the available evidence. Priority was given to diagnostic test accuracy, reducing mortality, immediate complications, and long-term adverse effects. Clinical heterogeneity was explored by comparing inclusion criteria and patient characteristics at baseline as well as clinical differences in the interventions and co-interventions.

### Development and updating of recommendations

For each PICO question, the following updating options were available: (1) the recommendation of the preceding version remains valid and requires no changes (“confirmed”); (2) the recommendation requires modification (“modified”); (3) the recommendation is no longer valid or required and is deleted; (4) a new recommendation needs to be developed (“new”). An interdisciplinary expert group of clinicians with expertise in thoracic surgery, cardiac surgery, trauma surgery, and acute care reviewed the body of evidence, drafted recommendations based on the homogeneity of clinical characteristics and outcomes, the balance between benefits and harms as well as their clinical expertise, and proposed grades of recommendation (Table 2). In the absence of eligible evidence, good practice recommendations were made based on clinical experience, data from studies with a lower level of evidence, and expert consensus in cases where the Guideline Group felt a statement was required due to the importance of the topic. These were not graded, and instead labelled as good (clinical) practice points (GPP). For GPPs, the strength of a recommendation is presented in the wording shown in Table 2.

**Table 2:** Grading of recommendations

Symbol	Grade of recommendation	Description	Wording (examples)
⇑⇑	A	strong recommendation	“use…”, “do not use…”
⇑	B	recommendation	“should use…”, “should not use…”
⇔	0	open recommendation	“consider using…”, “… can be considered”

### Consensus process

The Guideline Group finalised the recommendations during a web-based, structured consensus conference on 13 September 2021 via Zoom (Zoom, Version: 5.x [Software], Zoom Video Communications, Inc., San José, California, USA. https://zoom.us). A neutral moderator facilitated the consensus conference. Voting members of the Guideline Group were delegates of all participating professional organisations, including clinicians, emergency medical services personnel and nurses, while guideline methodologists attended in a supporting role. Members with a moderate, thematically relevant conflict of interest abstained from voting on recommendations, members with a high, relevant conflict of interest were not permitted to vote or participate in the discussion. Attempts to recruit patient representatives were unsuccessful. A member of the expert group presented recommendations. Following discussion, the Guideline Group refined the wording of the recommendations and modified the grade of recommendation as needed. Agreement with both the wording and the grade of recommendation was assessed by anonymous online voting using the survey function of Zoom. Abstentions were subtracted from the denominator of the agreement rate. Consensus strength was classified as shown in Table 3.

**Table 3:** Classification of consensus strength

Description	Agreement rate
strong consensus	> 95% of participants
consensus	> 75 to 95% of participants
majority approval	> 50 to 75% of participants
no approval	< 50% of participants

Recommendations were accepted if they reached consensus or strong consensus. For consensus recommendations with ≤ 95% agreement, diverging views by members of the Guideline Group were detailed in the background texts. Recommendations with majority approval were returned to the expert group for revision and further discussion at a subsequent consensus conference. Recommendations without approval were considered rejected.

### External review

During a four-week consultation phase, the recommendations and background texts were submitted to all participating professional organisations for review. Comments were collected using a structured review form. The results were then assessed, discussed and incorporated into the text by the guideline coordinator with the relevant author group.

The guideline was adopted by the executive board of the German Trauma Society on 17 January 2023.

### Quality assurance

The guideline recommendations were reviewed for consistency between guideline topic areas by the steering group. Where necessary, changes were made in collaboration with the clinical leads for all topic areas concerned. The final guideline document was checked for errors by the guideline chair and methodologist.

## Results

**Fig. 1:**
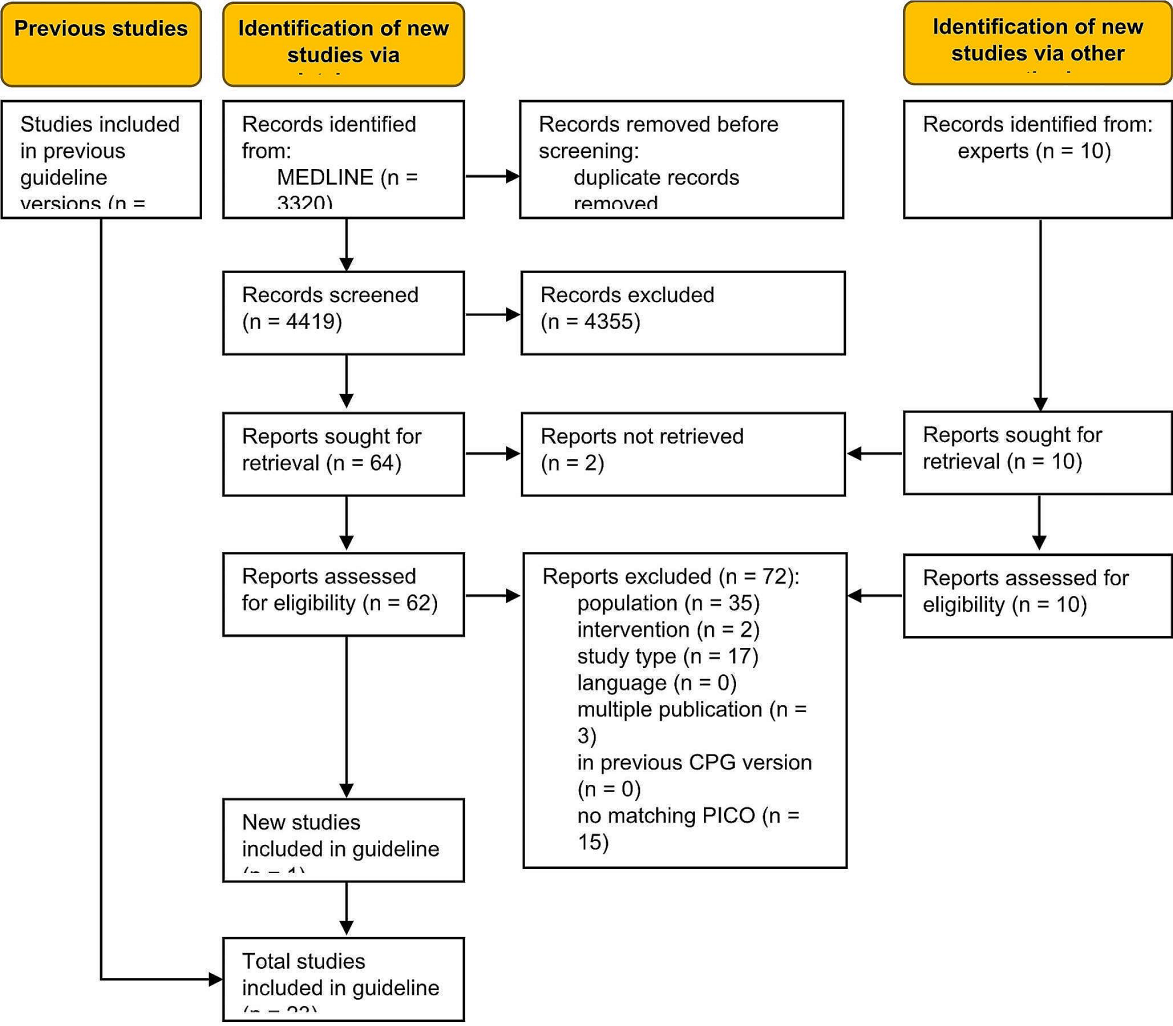
Modified PRISMA 2020 flow diagram showing the systematic literature search and selection of studies

The database searches identified 4419 unique records (Fig. 1). Ten additional records were obtained from clinical experts, adding to the body of evidence of 22 studies previously included in the guideline [12–33]. One study was eligible for this update [34]. A total of 72 full-text articles were excluded (Table S3, Online Resource 1).

### Characteristics of studies included in this update

Study characteristics, main outcomes, levels of evidence, and risk-of-bias assessments are presented in Table 4. Full details are provided in Table S4, Online Resource 1. The evidence included one comparative registry study [34]. Eligible patient populations were adults with chest trauma (defined as AIS ≥ 3) who required some form of lung resection.

**Table 4:** Characteristics of studies included in the update (see Table S4, Online Resource 1 for further details)

Study, reference	Design	Setting	Population	Age, ISS^*^	Interventions (*N* patients)	Main outcomes (selection)^*^	LoE, risk of bias (RoB)^§^
*Parenchyma-sparing surgery for lung injuries*							
Aiolfi 2020 [34]	Comparative registry study (National Trauma Data Bank)	2007–2015	Patients who sustained severe chest trauma	*Age [y], mean ± SD* 33 ± 16 *Mean ISS ± SD* 25 ± 13	*N* = 3107 IG: wedge resection (*N* = 1696) IG2: lobectomy (*N* = 1187) CG: pneumonectomy (*N* = 224)	*Overall mortality n (%)* IG: 344 (20.3) vs. IG2: 366 (30.8) vs. CG: 142 (63.4), *p* < 0.001	LoE 2b unclear RoB

* Data for IG versus CG unless otherwise specified. ^§^ Risk of bias: low RoB = RoB low for all domains; unclear RoB = RoB unclear for at least one domain, no high RoB in any domain; for studies with high RoB, all domains with high RoB are named, with RoB low or unclear for all other domains (for full details Table S4, Online Resource 1)

## Discussion

### Rationale for recommendations

The text below the recommendations discusses the various injuries and the surgical approach or surgical / interventional procedure. The therapeutic procedure and the surgical approach generally depend on the stability of the patient.

Surgical approach and indications for thoracotomy/thoracoscopy.

Depending on the localization of the injury, an anterolateral thoracotomy or a sternotomy can be chosen as the thoracic approach. If the location of injury is unclear, a clam shell incision can be considered. (GoR 0)

Video-assisted thoracoscopic surgery (VATS) can be used to gain thoracic access or to perform a surgical procedure in patients with cardiorespiratory stability. (GPP)

Depending on the type of trauma and the location of injury, there are different surgical approaches to emergency thoracotomy. Anterolateral thoracotomy on the injured side is the standard approach to emergency thoracotomy. When this approach is used, exposure is insufficient in approximately 20% of cases [35]. If the injury can be accurately localised before surgery, other approaches are recommended as well. A sternotomy is used for injuries to the aortic arch, the great vessels, the heart, and the ascending aorta, and a right (posterolateral) thoracotomy for injuries to the intrathoracic trachea. A supraclavicular approach with division of the clavicle provides exposure of the subclavian vessels [35–40]. Injuries to the posterior wall of the heart are better approached through an anterolateral thoracotomy.

Whereas video-assisted thoracoscopic surgery (VATS) is an unsuitable approach in patients with cardiorespiratory instability, it is increasingly preferred over the aforementioned open approaches in stable or stabilised patients. VATS allows injuries to the lung and diaphragm and other intrathoracic sources of bleeding to be safely identified and managed. It is regularly utilised for the evacuation of retained haemothoraces, usually in the post-acute setting [41–43]. VATS can also be used during thoracotomy as a hybrid procedure to explore areas that are difficult to visualise.

Thoracotomy can be performed in stable and unstable patients with initially high blood loss or ongoing relevant blood loss through a chest tube. (GPP)

Video-assisted thoracoscopic surgery (VATS) can be performed as an alternative to thoracotomy in patients with cardiopulmonary stability. (GPP)

For many decades, an initial chest tube output of > 1500 mL or a continued loss of 250 mL/hour for over four hours has been recommended as an indication for emergency thoracotomy in the acute management of chest trauma. This recommendation is based on a study that McNamara et al. published fifty years ago on the basis of lessons learned during the Vietnam conflict and other studies that have continued the recommendations of McNamara et al. and which were published twenty or thirty years ago [40, 44–46].

For this reason, the Guideline Group addressed the question of whether this recommendation still holds since the underlying figures were reported in studies with a low level of evidence. In recent decades, stabilisation measures as well as diagnostic and therapeutic options in the management of polytrauma patients have been considerably improved so that the quantity details that provided the basis for the recommendation on indications for thoracotomy were deleted and replaced by “initially high blood loss or ongoing relevant blood loss”. This modification allows patients who are stable or have been stabilised and show an initially high chest tube output, which can stop spontaneously, or ongoing blood loss to be managed in a far more differentiated manner. Diagnostic computed tomography (CT), therapeutic measures (if necessary), VATS, etc. can thus be performed.

Since 1990, VATS has been increasingly used in clinical practice as an alternative to thoracotomy in haemodynamically stable or stabilised trauma patients. Several studies have shown that VATS offers advantages when compared to thoracotomy [41–43, 47]. In a prospective randomised study from China, VATS was associated with significantly shorter operating times (*p* < 0.05), less intraoperative bleeding, and a lower postoperative drainage volume in the management of patients with penetrating thoracic trauma [47]. Likewise, VATS is presently the method of choice for the early management (within the first four days of trauma) of patients with retained haemothorax [48]. In spite of the absence of high-quality comparative studies, VATS is increasingly used as the preferred surgical technique in the treatment of haemodynamically stable or stabilised patients with thoracic trauma. For this reason, VATS is recommended as a GPP for the initial surgical management of patients with cardiopulmonary stability.

### Penetrating chest injuries

Apart from the above-mentioned indications for surgical intervention, the Guideline Group recommends that retained foreign bodies should only be removed under controlled conditions in the operating room (GoR B) in order to prevent secondary injuries or the uncontrolled release of any tamponade effect. Patients in a stable haemodynamic and respiratory condition undergo chest tube placement. Further treatment then depends on the results of the diagnostic procedures that must then be performed. The retained foreign body also helps to detect the depth of injury.

Injuries to the lung and the tracheobronchial system.

If surgery is indicated (for ongoing bleeding and/or persistent air leaks) in patients with lung injuries, a parenchyma-sparing approach should be used. (GoR B)

The recommendation to use a parenchyma-sparing technique in the management of lung injuries that require surgery for ongoing bleeding and/or parenchymal fistula [49–51] was confirmed by a retrospective analysis of data from the National Trauma Data Bank [34].

Aiolfi et al. [34] reported a significant increase (*p* < 0.001) in mortality as the extent of resection increased (wedge resection, 20.3%; lobectomy, 30.8%; pneumonectomy, 63.4%; *p* < 0.001). After propensity score analysis, the odds ratio (OR) for mortality was 1.42 (95% confidence interval, 1.26–1.71) in the lobectomy group and 4.16 (95% confidence interval, 2.84–6.07) in the pneumonectomy group. Similarly, anatomical resections were associated with higher complication rates, more mechanical ventilation days, and longer intensive care unit and inhospital lengths of stay [34].

The recommendations in the 2016 Guideline for the management of injuries to the tracheobronchial system remain unchanged. There is a paucity of high-quality literature on these rare and often life-threatening injuries. If a tracheobronchial injury is suspected, tracheobronchoscopy should be performed (GoR B**)** to confirm the diagnosis in patients with a pneumothorax that persists in spite of a correctly placed and properly functioning chest drain as well as in patients with subcutaneous emphysema or atelectasis. Penetrating injuries most commonly affect the cervical trachea. Blunt trauma usually involves the intrathoracic portion of the tracheobronchial system, especially the right main bronchus [52–55]. Moreover, injuries in the region of the posterior wall of the trachea can occur during tube intubation [24]. Traumatic tracheobronchial injuries are usually managed surgically. Conservative management can be attempted (GoR 0) to treat small localised bronchial tissue defects (< 1/3 of the circumference) with well adapted bronchial margins [52–55]. If indicated, traumatic tracheobronchial injuries should be surgically managed early after diagnosis (GoR B) since delayed treatment is associated with increased complication rates. Cervical injuries are usually approached through a collar incision and thoracic injuries through a right posterolateral thoracotomy [21]. Nonoperative management of iatrogenic tracheal injuries is often an option in patients with uncomplicated ventilation and patients with superficial or sufficiently covered tears [24]. Stents have no role in the management of tracheobronchial injuries.

### Injuries to the great vessels and the heart

If technically and anatomically possible, endovascular stent grafting should be preferred to open repair in the management of thoracic aortic ruptures. (GoR B)

The timing of aortic rupture management depends on the haemodynamic status of the patient at the time of admission. Patients who are haemodynamically unstable (as a result of an aortic injury) and patients in extremis must undergo immediate surgery [56]. The standard treatment of aortic rupture consists of aortic repair using a direct suture with aortic clamping and a variety of bypass techniques allowing for perfusion of the lower body and the spinal cord during clamping (left-heart bypass, Gott shunt, heart-lung machine) [17, 22].

In the management of patients who do not require immediate life-saving surgery, the use of stent grafts for aortic rupture is a minimally invasive and time-saving therapeutic option that minimises damage from the surgical approach [57]. Compared with other surgical techniques, endovascular stenting was reported to be associated with the same technical success rate and significantly lower rates of mortality, postoperative neurological deficits (paraplegia, stroke) and other complications [23, 27, 57]. Based on the available evidence, the recommendation in the 2016 Guideline for aortic stent grafting in the management of traumatic aortic injuries was confirmed.

In patients requiring no immediate intervention, the timing of the management of aortic injuries depends on concomitant injuries. In patients with concomitant traumatic brain injury, severe abdominal or skeletal injuries that require immediate surgical intervention and in elderly patients with serious cardiac and pulmonary comorbidities, aortic repair may be delayed until other life-threatening injuries have been managed or the patient has been stabilised. This approach does not have any disadvantages for the patient [40, 56, 58].

When surgery is not performed as an emergency, strict pharmacologic control of blood pressure (a systolic blood pressure around 100 mmHg and a pulse < 100) with beta-blockers and vasodilators is recommended [18, 58]. Comparative prospective studies on blood pressure control are not available. The European Society of Cardiology (ESC) Guidelines recommend that mean blood pressure should not exceed 80 mmHg [59].

### Injuries to the heart

As a result of the lack of evidence in the available literature, the Guideline Group did not submit any agreed recommendations on cardiac injuries.

The literature emphasises, however, that cardiac injuries are associated with high mortality and require urgent attention [37, 38]. Sternotomy is usually the primary approach to gain access to the heart in an emergency. If anterolateral thoracotomy is used for access in an unclear situation, the incision can be extended to a clam shell thoracotomy in an acute emergency setting. Rapid bleeding control is important after immediate relief of life-threatening cardiac tamponade. Atrial injuries can be repaired by using sutures after the involved atrial wall is clamped [21]. The control of bleeding in more extensive injuries with structural defects of the heart usually requires the use of a heart-lung machine. Ventricular lesions in particular often require the successful use of a heart-lung machine. In such a situation, temporary occlusion can be achieved with a Foley catheter balloon that is inflated in the ventricle.

### Injuries to the bony part of the thoracic wall (without the spine)

In polytrauma patients, these injuries are usually not managed during initial surgical procedures but at a later stage following patient stabilisation. The Guideline Group did not submit any agreed recommendations on these injuries.

### Limitations of the guideline

Patient values and preferences were sought but not received. The effect of this on the guideline is unclear, and there is a lack of research evidence on the effect of patient participation on treatment decisions or outcomes in the emergency setting.

### Risk-of-bias assessment for included studies and levels of evidence

The study included in the update showed an unclear risk of performance and detection bias. The level of evidence was not downgraded for any study.

### Recommendations

One recommendation was modified. Two additional good practice points were developed based on the updated evidence and expert consensus (Table 5. All achieved strong consensus. (Table S5, Online Resource 1).

GoR, grade of recommendation; GPP, good (clinical) practice point; VATS, video-assisted thoracoscopic surgery.

**Table 5:** List of recommendations with grade of recommendation and strength of consensus

No.	GoR	Evidence, consensus^a^	Recommendation	Status 2022	
*Key recommendations*					
1	0 ⇔	100%	Depending on the location of injury, anterolateral thoracotomy or sternotomy can be used to access the chest. If the location of injury is unclear, a clam shell incision can be used.	Confirmed	
2	GPP	100%	Video-assisted thoracoscopic surgery (VATS) can be used to gain thoracic access or to perform a surgical procedure in patients with cardiorespiratory stability.	**New**	
3	B ⇑	100%	In patients with penetrating chest injuries, retained foreign bodies should only be removed under controlled conditions in the operating room after thoracotomy.	Confirmed	
4	A ⇑⇑	100%	Patients with a penetrating chest injury that is the cause of haemodynamic instability use immediate exploratory thoracotomy.	Confirmed	
5	GPP	100%	Thoracotomy can be performed in stable and unstable patients with initially high blood loss or ongoing relevant blood loss through a chest tube.	**Modified**	
6	GPP	100%	Video-assisted thoracoscopic surgery (VATS) can be performed as an alternative to thoracotomy in patients with cardiopulmonary stability.	**New**	
7	B ⇑	100% [34]	If surgery is indicated (for ongoing bleeding and/or persistent air leaks) in patients with lung injuries, a parenchyma-sparing approach should be used.	Confirmed	
8	B ⇑	100%	If technically and anatomically possible, endovascular stent grafting should be preferred to open repair in the management of thoracic aortic ruptures.	Confirmed	
9	B ⇑	100%	If an injury to the tracheobronchial system is suspected on clinical grounds, tracheobronchoscopy should be performed to confirm the diagnosis.	Confirmed	
10	B ⇑	100%	Traumatic injuries to the tracheobronchial system should be surgically managed early after diagnosis.	Confirmed	
11	0 ⇔	100%	Conservative management can be attempted to treat localised injuries to the tracheobronchial system.	Confirmed	

## Data Availability

A full version of the guideline and its methods/evidence report are available online at https://register.awmf.org/de/leitlinien/detail/187-023.

## Abbreviations

AAST: American Association for the Surgery of Trauma
AIS: Abbreviated Injury Scale
AWMF: German Association of the Scientific Medical Societies
CG: Control group
ESC: European Society of Cardiology
GoR: Grade of recommendation
GPP: Good (clinical) practice point
IG: Intervention group
ISS: Injury Severity Score
LoE: Level of evidence
mL: Millilitre
n.r.: Not reported
OR: Odds ratio
PICO: Population, intervention, comparison, outcome
PRISMA: Preferred Reporting Items for Systematic Reviews and Meta-Analyses
RCT: Randomised controlled trial
RoB: Risk of bias
RR: Risk ratio
SBP: Systolic blood pressure
Unadj: Unadjusted
VATS: Video-assisted thoracoscopic surgery
Y: Years

## References

[1] Hoefer C, Lefering R. Arbeitskreis TraumaRegister Der Sektion Notfall- & intensivmedizin und Schwerverletztenversorgung. Jahresbericht 2022 des TraumaregistersDGU® Akademie Der Unfallchirurgie 2022. Available from: Jahresbericht 2022 des TraumaregistersDGU® Akademie der Unfallchirurgie.

[2] Schulz-Drost S, Merschin D, Gümbel D, Matthes G, Hennig FF, Ekkernkamp A, et al. Emergency department thoracotomy of severely injured patients: an analysis of the TraumaRegister DGU®. Eur J Trauma Emerg Surg. 2020;46:473–85. 31520155 10.1007/s00068-019-01212-3

[3] S3-Leitlinie Polytrauma/Schwerverletzten-Behandlung, Registernummer 187– 023. (2022), Version 3.0. https://www.awmf.org/leitlinien/detail/ll/187-023.html.

[4] Chen Y, Yang K, Marusic A, Qaseem A, Meerpohl JJ, Flottorp S, et al. A Reporting Tool for Practice guidelines in Health Care: the RIGHT Statement. Ann Intern Med. 2017;166(2):128–32. 10.7326/M16-1565. Epub 20161122. 27893062 10.7326/M16-1565

[5] Page MJ, McKenzie JE, Bossuyt PM, Boutron I, Hoffmann TC, Mulrow CD, et al. The PRISMA 2020 statement: an updated guideline for reporting systematic reviews. BMJ. 2021;372:n71. 10.1136/bmj.n71. 33782057 10.1136/bmj.n71 PMC8005924

[6] Arbeitsgemeinschaft der Wissenschaftlichen Medizinischen Fachgesellschaften (AWMF)-Ständige Kommission Leitlinien. AWMF-Regelwerk „Leitlinien. 2nd Edition 2020. http://www.awmf.org/leitlinien/awmf-regelwerk.html. Accessed 11 November 2021.

[7] Deutsche Gesellschaft für Unfallchirurgie e.V. (DGU). Leitlinienreport zur AWMF Leitlinie Polytrauma / Schwerverletzten-Behandlung, Registernummer 012–019. (2016). https://www.awmf.org/leitlinien/detail/ll/012-019.html. Accessed 21 March 2022.

[8] Gliklich R, Dreyer N, Leavy M, editors. Registries for Evaluating Patient Outcomes: A User’s Guide. Third edition. Two volumes. AHRQ Publication No. 13(14)-EHC111. Rockville, MD: Agency for Healthcare Research and Quality; April 2014. http://www.effectivehealthcare.ahrq.gov/registries-guide-3.cfm. Prepared by the Outcome DEcIDE Center [Outcome Sciences, Inc., a Quintiles company] under Contract No. 290 2005 00351 TO7.

[9] Ouzzani M, Hammady H, Fedorowicz Z, Elmagarmid A. Rayyan-a web and mobile app for systematic reviews. Syst Reviews. 2016;5(1):210. 10.1186/s13643-016-0384-4. Epub 20161205. 10.1186/s13643-016-0384-4 PMC5139140 27919275

[10] National Institute for Health and Care Excellence (NICE). The guidelines manual: Appendices B-I, Published: 30 November 2012. https://www.nice.org.uk/process/pmg6/resources/the-guidelines-manual-appendices-bi-2549703709. Last accessed March 21, 2022.

[11] OCEBM Levels of Evidence Working Group*. Oxford Centre for Evidence-based Medicine Levels of Evidence. (March 2009). https://www.cebm.ox.ac.uk/resources/levels-of-evidence/oxford-centre-for-evidence-based-medicine-levels-of-evidence-march-2009. Last accessed March 21, 2022. 2009.

[12] Asensio JA, Berne JD, Demetriades D, Chan L, Murray J, Falabella A, et al. One hundred five penetrating cardiac injuries: a 2-year prospective evaluation. J Trauma. 1998;44(6):1073–82. 10.1097/00005373-199806000-00022. PubMed PMID: 9637165. 9637165 10.1097/00005373-199806000-00022

[13] Asensio JA, Murray J, Demetriades D, Berne J, Cornwell E, Velmahos G, et al. Penetrating cardiac injuries: a prospective study of variables predicting outcomes. J Am Coll Surg. 1998;186(1):24–34. 10.1016/s1072-7515(97)00144-0. PubMed PMID: 9449597. 9449597 10.1016/s1072-7515(97)00144-0

[14] Athanasiou T, Krasopoulos G, Nambiar P, Coats T, Petrou M, Magee P, et al. Emergency thoracotomy in the pre-hospital setting: a procedure requiring clarification. Eur J Cardiothorac Surg. 2004;26(2):377–86. PubMed PMID: 15296900. 15296900 10.1016/j.ejcts.2004.03.016

[15] Ayed AK, Al-Shawaf E. Diagnosis and treatment of traumatic intrathoracic major bronchial disruption. Injury. 2004;35(5):494–9. 10.1016/j.injury.2003.08..014. PubMed PMID: 15081327. 15081327 10.1016/j.injury.2003.08.014

[16] Baguley CJ, Sibal AK, Alison PM. Repair of injuries to the thoracic aorta and great vessels: Auckland, New Zealand 1995–2004. ANZ J Surg. 2005;75(6):383-7. 10.1111/j.1445-2197.2005.03398.x. PubMed PMID: 15943721. 10.1111/j.1445-2197.2005.03398.x 15943721

[17] Cardarelli MG, McLaughlin JS, Downing SW, Brown JM, Attar S, Griffith BP. Management of traumatic aortic rupture: a 30-year experience. Ann Surg. 2002;236(4):465–9. 10.1097/00000658-200210000-00009. discussion 9–70. 12368675 10.1097/00000658-200210000-00009 PMC1422601

[18] Fabian TC, Davis KA, Gavant ML, Croce MA, Melton SM, Patton JH Jr. et al. Prospective study of blunt aortic injury: helical CT is diagnostic and antihypertensive therapy reduces rupture. Ann Surg. 1998;227(5):666– 76; discussion 76– 7. 10.1097/00000658-199805000-00007. PubMed PMID: 9605658; PubMed Central PMCID: PMCPMC1191343. 10.1097/00000658-199805000-00007 PMC1191343 9605658

[19] Kummer C, Netto FS, Rizoli S, Yee D. A review of traumatic airway injuries: potential implications for airway assessment and management. Injury. 2007;38(1):27–33. 10.1016/j.injury.2006.09.002. Epub 20061031. 17078954 10.1016/j.injury.2006.09.002

[20] Martin MJ, McDonald JM, Mullenix PS, Steele SR, Demetriades D. Operative management and outcomes of traumatic lung resection. J Am Coll Surg. 2006;203(3):336–44. 10.1016/j.jamcollsurg.2006.05.009. Epub 20060711. 16931306 10.1016/j.jamcollsurg.2006.05.009

[21] Meredith JW, Hoth JJ. Thoracic trauma: when and how to intervene. Surg Clin North Am. 2007;87(1):95–118, vii. 10.1016/j.suc.2006.09.014. PubMed PMID: 17127125. 10.1016/j.suc.2006.09.014 17127125

[22] Miller PR, Kortesis BG, McLaughlin CA 3rd, Chen MY, Chang MC, Kon ND, et al. Complex blunt aortic injury or repair: beneficial effects of cardiopulmonary bypass use. Ann Surg. 2003;237(6):877–83. 0. PubMed PMID: 12796585; PubMed Central PMCID: PMCPMC1514682. 12796585 10.1097/01.SLA.0000071566.43029.E0 PMC1514682

[23] Peterson BG, Matsumura JS, Morasch MD, West MA, Eskandari MK. Percutaneous endovascular repair of blunt thoracic aortic transection. J Trauma. 2005;59(5):1062–5. 10.1097/01.ta.0000188634.72008.d5. PubMed PMID: 16385279. 16385279 10.1097/01.ta.0000188634.72008.d5

[24] Schneider T, Storz K, Dienemann H, Hoffmann H. Management of iatrogenic tracheobronchial injuries: a retrospective analysis of 29 cases. Ann Thorac Surg. 2007;83(6):1960–4. 10.1016/j.athoracsur.2007.01.042. PubMed PMID: 17532378. 17532378 10.1016/j.athoracsur.2007.01.042

[25] Schneider T, Volz K, Dienemann H, Hoffmann H. Incidence and treatment modalities of tracheobronchial injuries in Germany. Interact Cardiovasc Thorac Surg. 2009;8(5):571–6. 10.1510/icvts.2008.196790. Epub 20090211. 19211582 10.1510/icvts.2008.196790

[26] Symbas PN, Sherman AJ, Silver JM, Symbas JD, Lackey JJ. Traumatic rupture of the aorta: immediate or delayed repair? Ann Surg. 2002;235(6):796–802. 10.1097/00000658-200206000-00006. PubMed PMID: 12035035; PubMed Central PMCID: PMCPMC1422508. 12035035 10.1097/00000658-200206000-00006 PMC1422508

[27] Tang GL, Tehrani HY, Usman A, Katariya K, Otero C, Perez E, et al. Reduced mortality, paraplegia, and stroke with stent graft repair of blunt aortic transections: a modern meta-analysis. J Vasc Surg. 2008;47(3):671–5. 10.1016/j.jvs.2007.08.031. Epub 20071105. 17980541 10.1016/j.jvs.2007.08.031

[28] Demetriades D, Velmahos GC, Scalea TM, Jurkovich GJ, Karmy-Jones R, Teixeira PG, et al. Blunt traumatic thoracic aortic injuries: early or delayed repair–results of an American Association for the surgery of Trauma prospective study. J Trauma. 2009;66(4):967–73. 10.1097/TA.0b013e31817dc483. PubMed PMID: 19359900. 19359900 10.1097/TA.0b013e31817dc483

[29] Inaba K, Lustenberger T, Recinos G, Georgiou C, Velmahos GC, Brown C, et al. Does size matter? A prospective analysis of 28–32 versus 36–40 French chest tube size in trauma. J Trauma Acute Care Surg. 2012;72(2):422–7. 10.1097/TA.0b013e3182452444. PubMed PMID: 22327984. 22327984 10.1097/TA.0b013e3182452444

[30] Kirkpatrick AW, Rizoli S, Ouellet JF, Roberts DJ, Sirois M, Ball CG, et al. Occult pneumothoraces in critical care: a prospective multicenter randomized controlled trial of pleural drainage for mechanically ventilated trauma patients with occult pneumothoraces. J Trauma Acute Care Surg. 2013;74(3):747–54. 10.1097/TA.0b013e3182827158. discussion 54– 5. 23425731 10.1097/TA.0b013e3182827158

[31] Ouellet JF, Trottier V, Kmet L, Rizoli S, Laupland K, Ball CG, et al. The OPTICC trial: a multi-institutional study of occult pneumothoraces in critical care. Am J Surg. 2009;197(5):581–6. 10.1016/j.amjsurg.2008.12.007. PubMed PMID: 19306978. 19306978 10.1016/j.amjsurg.2008.12.007

[32] Yadav K, Jalili M, Zehtabchi S. Management of traumatic occult pneumothorax. Resuscitation. 2010;81(9):1063–8. 10.1016/j.resuscitation.2010.04.030. PubMed PMID: 20619952. 20619952 10.1016/j.resuscitation.2010.04.030

[33] Yi JH, Liu HB, Zhang M, Wu JS, Yang JX, Chen JM, et al. Management of traumatic hemothorax by closed thoracic drainage using a central venous catheter. J Zhejiang Univ Sci B. 2012;13(1):43–8. 10.1631/jzus.B1100161. PubMed PMID: 22205619; PubMed Central PMCID: PMCPMC3251751. 22205619 10.1631/jzus.B1100161 PMC3251751

[34] Aiolfi A, Inaba K, Martin M, Matsushima K, Bonitta G, Bona D, et al. Lung resection for trauma: a propensity score adjusted analysis comparing Wedge Resection, Lobectomy, and Pneumonectomy. Am Surg. 2020;86(3):261–5. PubMed PMID: rayyan-671314007. 32223808

[35] Karmy-Jones R, Nathens A, Jurkovich GJ, Shatz DV, Brundage S, Wall MJ Jr. et al. Urgent and emergent thoracotomy for penetrating chest trauma. J Trauma. 2004;56(3):664-8; discussion 8–9. 10.1097/01.ta.0000068238.74552.4b. PubMed PMID: 15128141. 10.1097/01.ta.0000068238.74552.4b 15128141

[36] Branney SW. Moore Ee Fau - Feldhaus KM, Feldhaus Km Fau - Wolfe RE, Wolfe RE. Critical analysis of two decades of experience with postinjury emergency department thoracotomy in a regional trauma center. 1998;(0022-5282 (Print)). 10.1097/00005373-199807000-00019 9680018

[37] Feliciano DV, Rozycki GS. Advances in the diagnosis and treatment of thoracic trauma. Surg Clin North Am. 1999;79(6):1417–29. 10.1016/S0039-6109(05)70085-2. 10625986 10.1016/s0039-6109(05)70085-2

[38] Wall MJ, Soltero E. Damage control for thoracic injuries. Surg Clin North Am. 1997;77(4):863–78. 10.1016/S0039-6109(05)70590-9. 9291987 10.1016/s0039-6109(05)70590-9

[39] Xenos ES, Freeman M, Stevens S, Cassada D, Pacanowski J, Goldman M. Covered stents for injuries of subclavian and axillary arteries. J Vasc Surg. 2003;38(3):451–4. 10.1016/s0741-5214(03)00553-6. PubMed PMID: 12947252. 12947252 10.1016/s0741-5214(03)00553-6

[40] Wall MJ, Hirshberg A, LeMaire SA, Holcomb J, Mattox K. Thoracic aortic and thoracic vascular injuries. Surg Clin North Am. 2001;81(6):1375–93. 10.1016/S0039-6109(01)80013-X. 11766181 10.1016/s0039-6109(01)80013-x

[41] Ben-Nun A, Orlovsky M, Best LA. Video-assisted thoracoscopic surgery in the treatment of chest trauma: long-term benefit. Ann Thorac Surg. 2007;83(2):383–7. 10.1016/j.athoracsur.2006.09.082. 17257954 10.1016/j.athoracsur.2006.09.082

[42] Goodman M, Lewis J, Guitron J, Reed M, Pritts T, Starnes S. Video-assisted thoracoscopic surgery for acute thoracic trauma. J Emerg Trauma Shock. 2013;6(2):106–9. 10.4103/0974-2700.110757. PubMed PMID: 23723618; PubMed Central PMCID: PMCPMC3665056. 23723618 10.4103/0974-2700.110757 PMC3665056

[43] Billeter AT, Druen D, Franklin GA, Smith JW, Wrightson W, Richardson JD. Video-assisted thoracoscopy as an important tool for trauma surgeons: a systematic review. Langenbecks Arch Surg. 2013;398(4):515–23. 10.1007/s00423-012-1016-7. Epub 20130404. 23553352 10.1007/s00423-012-1016-7

[44] McNamara JJ, Messersmith JK, Dunn RA, Molot MD, Stremple JF. Thoracic injuries in Combat casualties in Vietnam. Ann Thorac Surg. 1970;10(5):389–401. 10.1016/S0003-4975(10)65367-2. 5476227 10.1016/s0003-4975(10)65367-2

[45] Karmy-Jones R, Jurkovich Gj Fau - Nathens AB. Nathens Ab Fau - Shatz DV, Shatz Dv Fau - Brundage S, Brundage S Fau - Wall MJ, Jr., Wall Mj Jr Fau - Engelhardt S, Timing of urgent thoracotomy for hemorrhage after trauma: a multicenter study. 2001;(0004–0010 (Print)). 10.1001/archsurg.136.5.513 11343541

[46] Mansour MA, Moore Ee Fau -, Moore FA, Moore Fa Fau - Read RR, Read RR. Exigent postinjury thoracotomy analysis of blunt versus penetrating trauma. 1992;(0039-6087 (Print)). 1636147

[47] Jin J, Song B, Lei YC, Leng XF. Video-assisted thoracoscopic surgery for penetrating thoracic trauma. Chin J Traumatol. 2015;18(1):39–40. PubMed PMID: 26169093. 26169093 10.1016/j.cjtee.2014.07.003

[48] Patel NJ, Dultz L, Ladhani HA, Cullinane DC, Klein E, McNickle AG, et al. Management of simple and retained hemothorax: a practice management guideline from the Eastern Association for the surgery of Trauma. Am J Surg. 2021;221(5):873–84. PubMed PMID: 33487403. 33487403 10.1016/j.amjsurg.2020.11.032

[49] Cothren C, Moore E, Biffl W, Franciose R, Offner P, Burch J. Lung-sparing techniques are associated with improved outcome compared with anatomic resection for severe lung injuries. J Trauma. 2002;53(3):483–7. 10.1097/01.TA.0000025399.13825.35. 12352485 10.1097/00005373-200209000-00015

[50] Gasparri M, Karmy-Jones R, Kralovich K, Patton JJ, Arbabi S. Pulmonary tractotomy versus lung resection: viable options in penetrating lung injury. J Trauma. 2001;51(6):1092–5. 11740259 10.1097/00005373-200112000-00013

[51] Huh J, Wall MJ, Estrera A, Soltero E, Mattox K. Surgical management of traumatic pulmonary injury. Am J Surg. 2003;186(6):620–4. 10.1016/S0002-9610(03)00392-1. 14672768 10.1016/j.amjsurg.2003.08.013

[52] Balci A, Eren N, Eren S, Ulku R. Surgical treatment of post-traumatic tracheobronchial injuries: 14-year experience. Eur J Cardiothorac Surg. 2002;22(6):984–9. 12467824 10.1016/s1010-7940(02)00591-2

[53] Kiser AC, O’Brien SM, Detterbeck FC. Blunt tracheobronchial injuries: treatment and outcomes. Ann Thorac Surg. 2001;71(6):2059–65. 10.1016/S0003-4975(00)02453-X. 11426809 10.1016/s0003-4975(00)02453-x

[54] Rossbach MM, Johnson SB, Gomez MA, Sako EY, Miller OL, Calhoon JH. Management of Major Tracheobronchial injuries: a 28-Year experience. Ann Thorac Surg. 1998;65(1):182–6. 10.1016/S0003-4975(97)01001-1. 9456114 10.1016/s0003-4975(97)01001-1

[55] Dienemann H, Hoffmann H. Tracheo-Bronchiale Verletzungen Und Fisteln. Chirurg. 2001;72:1130–6. 10.1007/s001040170050 11715615

[56] Camp PC, Shackford SR. Outcome after Blunt traumatic thoracic aortic laceration: identification of a high-risk cohort. J Trauma Acute Care Surg. 1997;43(3):413–22. PubMed PMID: 00005373-199709000-00004. 10.1097/00005373-199709000-00004 9314301

[57] Ott MC, Stewart TC, Lawlor DK, Gray DK, Forbes TL. Management of blunt thoracic aortic injuries: endovascular stents versus open repair. J Trauma. 2004;56(3):565–70. 10.1097/01.ta.0000114061.69699.a3. PubMed PMID: 15128128. 15128128 10.1097/01.ta.0000114061.69699.a3

[58] Fabian TC, Richardson JD, Croce MA, Smith JS, Rodman G, Kearney PA, et al. Prospective study of Blunt Aortic Injury: Multicenter Trial of the American Association for the surgery of Trauma. J Trauma Acute Care Surg. 1997;42(3):374–83. PubMed PMID: 00005373-199703000-00003. 10.1097/00005373-199703000-00003 9095103

[59] Erbel R, Fau - Aboyans V, Aboyans V, Fau - Boileau C, Boileau C, Fau - Bossone E, Bossone E, Fau - Bartolomeo RD, Bartolomeo Rd Fau -, Eggebrecht H, Eggebrecht H, Fau - Evangelista A, et al. 2014 ESC guidelines on the diagnosis and treatment of aortic diseases: document covering acute and chronic aortic diseases of the thoracic and abdominal aorta of the adult. The Task Force for the diagnosis and treatment of aortic diseases of the European Society of Cardiology (ESC). Eur Heart J. 2014;35(41):1522–9645. (Electronic). 10.1093/eurheartj/ehu281 25173340

